# Beyond Efficiency: Considering the Benefits of Residents in the Emergency Department

**DOI:** 10.5811/westjem.41505

**Published:** 2025-05-30

**Authors:** Jake Valentine, Johnathan Poulson

**Affiliations:** * University of Houston, Department, Houston, Texas; † HCA Houston Healthcare, Department, Kingwood, Texas

Dear Dr. Armstrong:

Thank you for your thoughtful comments and interest in our article: “Impact of Medical Trainees on Efficiency and Productivity in the Emergency Department: Systematic Review and Narrative Synthesis.”

We appreciate your perspective on the benefits that emergency medicine residents bring to the ED. In our review, we deliberately excluded papers on the impact of trainees on patient satisfaction and patient outcomes. We screened many interesting articles that addressed these topics and believe they merit their own separate systematic reviews.

We agree that there are numerous considerations when starting or hosting a residency program within an ED. One of the core tenets of competency-based medical education is aligning educational outputs with community needs. Ideally, this would involve demonstrating a need for more emergency physicians in the geographic area of the proposed residency and showing that graduates are staying to provide care in these underserved communities. Unfortunately, there is currently no accrediting body that considers this alignment. The Accreditation Council for Graduate Medical Education’s scope is limited to ensuring the adequacy of training rather than assessing the necessity of a program based on community needs.

Finally, we would also caution against relying on “intangible” measures of trainee value. As researchers, we are tasked with exploring these variables to provide evidence-based insights. Our concern is that accepting the benefits of academia as “intangible” may contribute to continued non-competitive compensation practices by academic institutions. As highlighted recently, the market often undervalues additional credentials and fellowship training, leading to large pay disparities between academic and community physicians.^1^

Once again, thank you for your engaging comments. We value the opportunity to discuss these important issues further.

Sincerely,

Jonathan Poulson DO, MSBME

Jake Valentine, MD, MEd
